# Acute Lymphocytic Myocarditis With Mobile Left Ventricular Thrombus

**DOI:** 10.7759/cureus.47262

**Published:** 2023-10-18

**Authors:** Ryosuke Honda, Toru Miyoshi, Shuntaro Ikeda, Hironori Izutani, Osamu Yamaguchi

**Affiliations:** 1 Department of Cardiology, Pulmonology, Hypertension, and Nephrology, Ehime University Graduate School of Medicine, Toon City, JPN; 2 Department of Cardiovascular Surgery, Ehime University Graduate School of Medicine, Toon City, JPN

**Keywords:** surgical thrombectomy, left ventricular thrombus, acute heart failure, acute myocarditis, lymphocytic myocarditis

## Abstract

A left ventricular thrombus (LVT) in cardiac disease is associated with various adverse events. To understand the risk of thromboembolism, it is necessary to recognize when LVT is most likely to occur.

We present a case of acute lymphocytic myocarditis in a young patient, where the LVT manifestation occurred within 48 hours after the beginning of the disease and was treated by thrombectomy. We have not found any reports of LVT formation earlier than in this case.

Patients with acute myocarditis are younger, at a median age of 34 years, and the social impact of embolic complications is considerable. Echocardiography is effective in detecting LVT noninvasively in low- and high-risk patients. Patients with myocarditis can benefit from careful observation by echocardiography.

## Introduction

A left ventricular thrombus (LVT) is occasionally observed in ischemic and nonischemic cardiomyopathy [1]. LVT is associated with up to a 37% risk of major adverse cardiovascular events, including death, stroke, myocardial infarction, or acute peripheral artery embolism [2].

It must be detected as soon as possible to initiate treatment early and prevent complications. However, there is no consensus regarding the timing of LVT complications in acute myocarditis. We present a case of acute lymphocytic myocarditis in a young patient, where the LVT manifestation occurred within 48 hours after the beginning of the disease and was treated by thrombectomy.

## Case presentation

**Figure 1:**
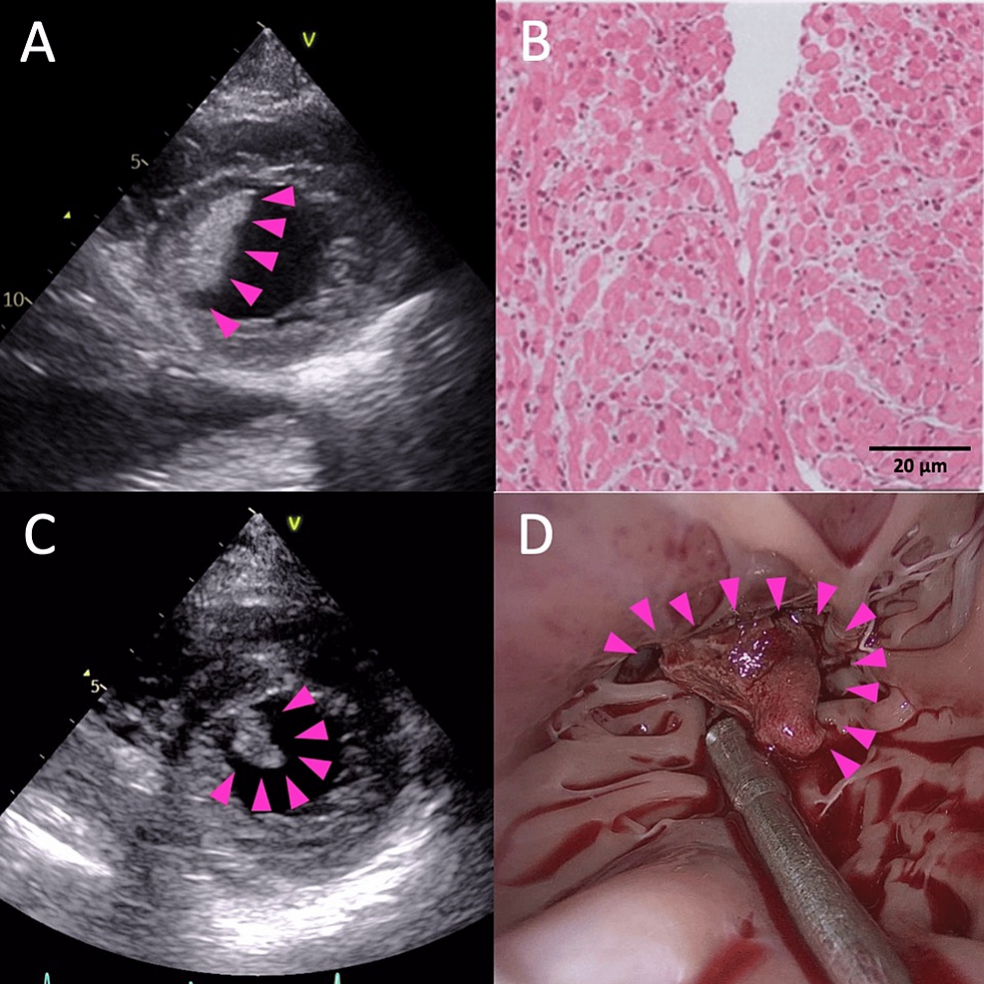
Left ventricular thrombus. A: Left ventricular thrombus in apex detected from parasternal short axis view of transthoracic echocardiography. B: Lymphocyte infiltration in myocardial tissue. C: Protrusion of the thrombus. D: Left ventricular thrombectomy also showed a thrombus protruding and easily detached.

A 24-year-old woman was accepted into our hospital and stayed for two days due to fever, fatigue, and chest discomfort. She had no past medical history of heart disease and was on no medication, including oral contraceptives. She was in cardiogenic shock upon admission with a blood pressure of 90/60 mmHg and a heart rate of 120 bpm. Laboratory examination abnormalities included a lactate of 2.1 mmol/l, brain natriuretic peptide of 511 pg/ml, and troponin I of 844.9 pg/ml. The blood eosinophil count was within normal range and the SARS-CoV-2 antigen test was negative. On transthoracic echocardiography, the left ventricular end-diastolic diameter (LVDd) was 52 mm, the left ventricular ejection fraction was 20% with diffuse severe hypokinesis, and a 30 × 20 mm-sized thrombus was detected at the left ventricular (LV) apex (Figure 1A).

Dobutamine was started at 5 mcg/kg/min and the patient underwent cardiac catheterization immediately. The coronary arteries had no significant stenosis; therefore, gamma-globulin, corticosteroids, and warfarin were administered before the pathology results were available.

Endomyocardial biopsy revealed CD3-positive T-cell infiltration with myocardial cell injury without eosinophils or giant cells, thus proving the diagnosis of acute lymphocytic myocarditis (Figure 1B).

The viral screening was all negative, and the patient did not have obvious coagulopathy, vasculitis, autoimmune disorders, or COVID-19.

The LV wall’s motion gradually improved, and on the 10th day, the thrombus shrank to 16 × 11 mm with a stalk and became mobile (Figure 1C).

We decided to perform the LV thrombectomy (Figure 1D). The thrombus mainly comprised a fibrin network, and the blood cell component was mostly erythrocytes with some neutrophils but no eosinophils.

Without postoperative complications, the patient was discharged on the 24th day without any embolic events.

## Discussion

Herein, LVT was found within a short period of 48 hours after the onset of acute lymphocytic myocarditis.

The LVT occurred as a result of the interplay of three factors: stasis attributable to reduced ventricular dysfunction, endocardial injury, inflammation, or hypercoagulability in a patient with heart disease [3]. The incidence of thromboembolic events in patients with systolic heart failure ranges from 1.5% to 2.7% per year [1], ischemic heart disease accounts for 80%, and dilated cardiomyopathy for 8% of these events [4]. However, the incidence of LVT in patients with acute myocarditis is not well known. One study reported that 62% of patients with acute myocarditis were diagnosed with LVT by cardiac endoscopy [5].

To the best of our knowledge, there have been no reports of massive LVT detected in such a short time, as we experienced in our case. Oberoi et al. report that LVT was noted at three weeks from *Mycoplasma pneumoniae* infection, and the autoimmune mechanism was considered to be the most likely cause [6]. In the case reported by Van Dam et al., five days had passed since the onset of myocarditis [7]. We have not found any reports of LVT formation earlier than in this case.

LVT in acute myocarditis may increase the risk of embolism as LV wall motion improves over time, in contrast to myocardial infarction and dilated cardiomyopathy. In addition, patients with acute myocarditis are younger, at a median age between 30 and 45 years [8], and the social impact of embolic complications is considerable. Surgical thrombectomy is one treatment option [9].

The LVT was identified early by echocardiography, and the thrombectomy was performed to prevent embolic complications.

## Conclusions

Acute lymphocytic myocarditis can be associated with LV thrombus formation. Echocardiography is effective in detecting LVT noninvasively in low- and high-risk patients. Patients in the early phase of acute myocarditis can benefit from careful observation by echocardiography, as LVT can form in the first 48 hours after the onset of the disease. Thrombectomy is successful after the precise diagnosis of thrombus using echocardiography.
